# Regulation of osteogenesis and osteoclastogenesis by zoledronic acid loaded on biodegradable magnesium-strontium alloy

**DOI:** 10.1038/s41598-018-37091-8

**Published:** 2019-01-30

**Authors:** Mei Li, Peng Wan, Weidan Wang, Ke Yang, Yu Zhang, Yong Han

**Affiliations:** 1 State Key Laboratory for Mechanical Behavior of Materials, Xi’an Jiaotong University, Xi’an, 710049 China; 2 Department of Orthopedics, Guangdong General Hospital, Guangdong Academy of Medical Sciences, Guangzhou, 510080 China; 3 School of Mechanical Engineering, Dongguan University of Technology, Dongguan, 523808 China; 4 Institute of Metal Research, Chinese Academy of Sciences, Shenyang, 110016 China; 5 University of Chinese Academy of Sciences, Beijing, 100049 China

## Abstract

Inhibiting osteoclasts and osteoclast precursors to reduce bone resorption is an important strategy to treat osteoclast-related diseases, such as peri-prosthetic osteolysis. In this study, our objective was to study the role of zoledronic acid (ZA), as a highly potent and nitrogen-containing bisphosphonate, in promoting osteogenesis and inhibiting osteoclastogenesis properties of magnesium (Mg)-based implants. ZA was chemically associated with calcium phosphate (CaP) deposited on magnesium-strontium (Mg-Sr) alloy, which was confirmed by the morphological observation, phase composition and drug releasing via SEM, XRD spectrum and High Performance Liquid Chromatography (HPLC), respectively. The *in vitro* performances indicated that ZA-CaP bilayer coating Mg-Sr alloy could enhance the proliferation and the osteogenic differentiation as well as the mineralization of pre-osteoblasts, however, induce the apoptosis and inhibit the osteoclast differentiation. We further investigated the possible molecular mechanisms by using Quantitative real-time PCR (qRT-PCR) and Western Blotting, and the results showed that ZA-CaP bilayer coating Mg-Sr alloy could regulate the osteogenesis and osteoclastogenesis through the Estrogen Receptor α (ERα) and NF-κB signaling pathway. Moreover, ZA-CaP bilayer coating Mg-Sr alloy could regulate the cross talk of osteoblast-osteoclast and increase the ratio of OPG: RANKL in the co-culture system through OPG/RANKL/RANK signaling pathway, which promoting the balance of bone remodeling process. Therefore, these promising results suggest the potential clinical applications of ZA pretreated Mg-Sr alloys for bone defect repairs and periprosthetical osteolysis due to the excessive differentitation and maturation of osteoclasts.

## Introduction

Biodegradable magnesium (Mg) and Mg alloys combine the superiorities of metallic and biodegradable implants, including the low specific density, high mechanical property and good compatibility^1^, which make them suitable for use as orthopedic biomaterials. It is also expected that the bone reconstruction risk of stress shielding and hardware failure may be reduced due to the relatively low elastic modulus of the alloys. Moreover, the released appropriate Mg ions could regulate signaling pathways of bone marrow stromal cells and stimulate new bone formation^2^. Clinically, a prospective study of MAGNEZIX^®^ Mg screws (Syntellix AG, Hannover, Germany) demonstrated that they functioned equivalently to titanium screws during the slight hallux valgus deformities healing^3^. However, rapid and continuous degradation may reduce the mechanical integrity and support properties of Mg implants. Therefore, regulating the degradation rate is crucial to the applications of Mg implants^4^. There has been considerable effort to enhance osseointegration between bone and Mg implants, such as the protective coating generated on Mg alloys^5^. To some extent, the single or composite coatings could reduce the degradation and enhance the corrosion resistance of Mg implants *in vitro* or *in vivo*^6,7^.

Bone regeneration of defects relies on the bidirectional balance between osteogenesis and osteoclastogenesis, wherein osteoblasts and osteoclasts play central roles^8,9^. Generally, inclining the balance beneficial to the differentiation of osteoclasts results in excessive bone absorption, as seen in peri-implant osteolysis, which remains the major reason for long-term failure and bad loading capacity of orthopedic implants^10,11^. Such a phenomenon is not uncommon clinically, especially in patients with osteoporosis. Encouraged formation of osseointegration around the bone implants and reduced generation of osteolysis surrounding prosthesis are of great importance to reinforce bone-implant fixation^12^. It is thereby of great clinical significance to develop new materials with therapeutic targeting of osteoclast function for treating or alleviating osteolysis. Actually, many drugs with the properties of anti-osteolysis have been widely investigated in the past several decades, such as bisphosphonates, which is the most common choice for the treatment of osteoporosis by inhibiting osteoclasts activity^13^. Zoledronic acid (ZA), as a long-acting bisphosphonate, could be given as an annual intravenous infusion and increase the callus volume significantly^14^. However, considering the undesirable effects such as gastrointestinal irritation, osteonecrosis of jaw and impairment of renal function in systemic use^15^, local application of ZA, directly targeting of the location where osteoclast action, seems more effective. Regarding the application potential of Mg-Sr alloy as bone substitutes and the advantages of ZA as an effective therapy to reduce osteolysis^16^, we prepared a novel bilayer coating on Mg-Sr alloy by the deposition of CaP and ZA, and investigated whether local delivery of ZA from Mg based bone implants might be effective for osteogenesis and osteoclastogenesis in our present study. We undertook this investigation: (1) to evaluate the possibility for fast loading by soaking of CaP coating Mg alloys into ZA solution; (2) to describe the release of ZA from novel Mg alloys and its elution dynamics; (3) to determine the local osteogenesis promoting and osteolysis suppression efficacy of ZA coating Mg-Sr alloy *in vitro* and (4) to illuminate the potential molecular mechanisms.

## Results

### Coating characterization

**Figure 1:**
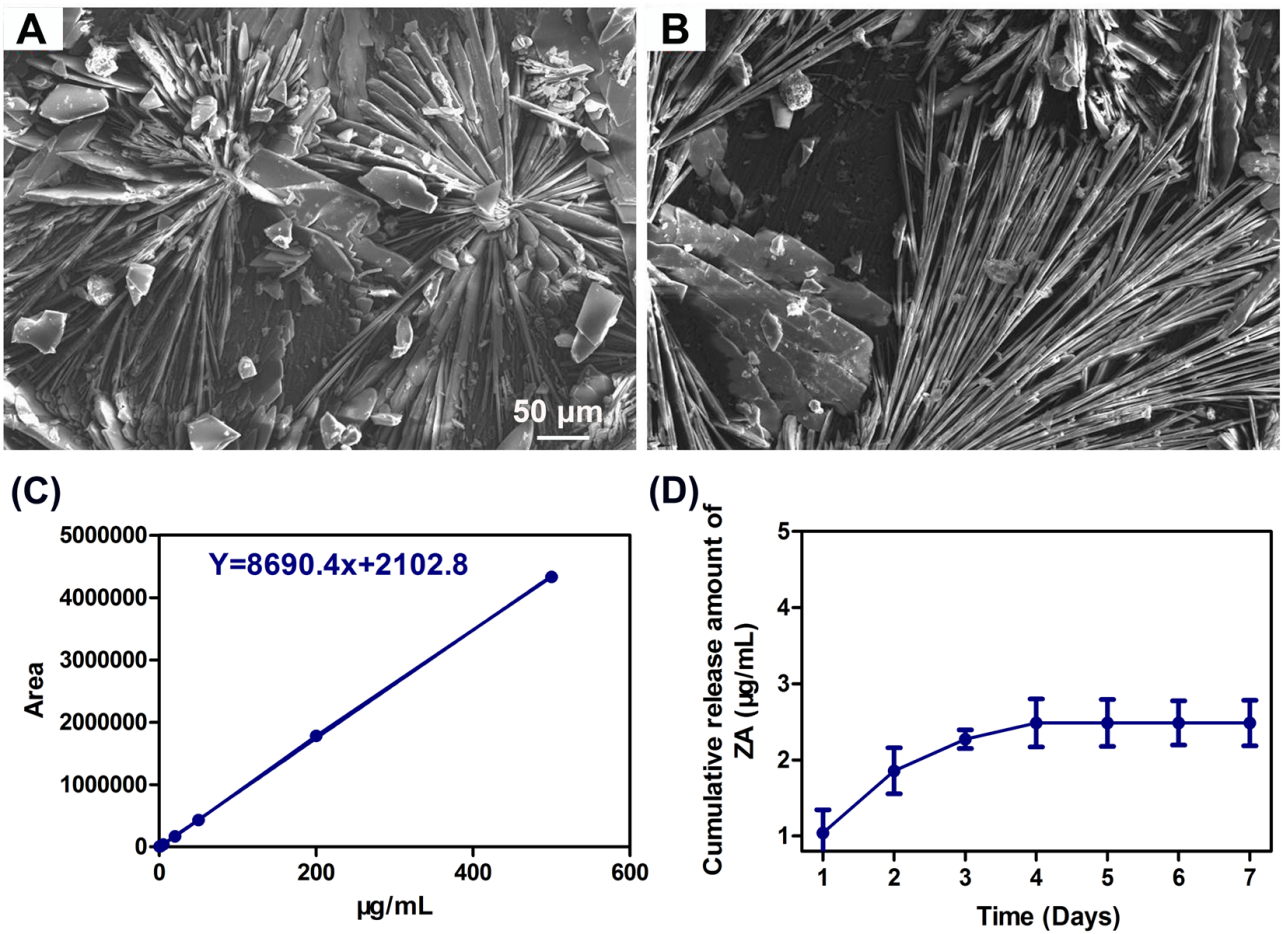
SEM micrographs performed on the Ca-P coating grown on the surface of Mg-Sr alloys before (**A**) and after (**B**) the treatment with 10^−4^ M of ZA. The calibration curve of pure ZA solution (**C**) and cumulative amount of ZA released from ZA-CaP bilayer coating Mg-Sr alloys after 1 to 7 days by HPLC (**D**).

Figure 1 shows the morphologies of the coatings grown on Mg-1.5wt.%Sr substrate before (CaP coating) and after (ZA-CaP coating) a treatment with 10^−4^ mol/L ZA solution. The CaP coating exhibits typical block-like crystalline structure (Fig. 1A). There is no visible modification in the morphology of crystallites structure after incorporating with ZA. However, well-arranged spicule microcrystallites start to form on the bilayer ZA-CaP coating Mg-Sr alloy (Fig. 1B). The refinement of crystallization can be ascribed to the calcium depletion in the presence of ZA solutions, and the partial dissolution of CaP layer on the surface of Mg alloy further induces the re-precipitation of surface coatings^17^. Figure 1C presents that the calibration curve of of pure ZA solution is linear within the concentration range of 0.2 to 500 µg/mL (R^2^ > 0.999), and the release of ZA from bilayer coating Mg-Sr alloy is greatest during the first twenty-four hours (1.04 µg/mL) and decrease rapidly during the next forty-eight hours to reach a plateau after four days (Fig. 1D). The highest amount of cumulative released ZA reaches to 2.485 µg/mL (8.56 µM) in 7 days. The concentrations of Mg and Sr ions releasing after immersion of 1, 3, 5 days in the cell culture medium can be seen in Fig. S1.

**Figure 2:**
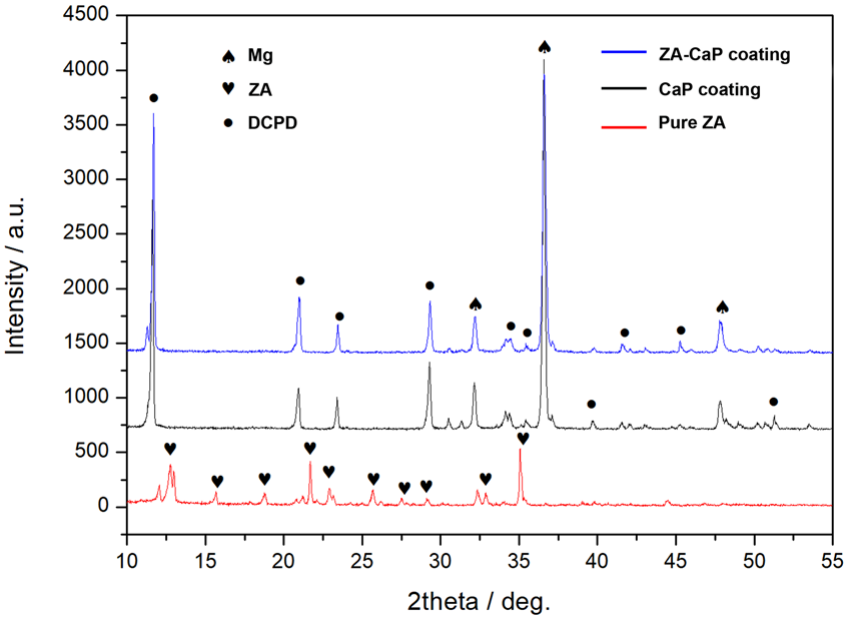
XRD spectra of Ca-P coating on Mg-Sr alloys before and after the incorporation of 10^−4^ M of ZA. Pure ZA powder was used as reference. A large number of Mg reflections and the characteristic peaks attributed to CaHPO_4_·2H_2_O (DCPD) can be detected on CaP and ZA-CaP coating Mg-Sr alloys.

Figure 2 illustrates the X-ray diffractometer (XRD) patterns for the CaP coatings before and after incorporating with ZA, and we set the profile of pure ZA as the reference pattern. After forming the CaP monolayer coating on Mg-1.5%Sr alloys, it is possible to detect a large number of Mg reflections and the characteristic peaks attributed to CaHPO_4_·2H_2_O (DCPD). However, the peaks of ZA cannot be observed in the diffraction pattern for ZA-CaP bilayer coating due to such low amounts (10^−4^ mol/L) of the drug.

### Cyto-compatibility of pre-osteoblasts

**Figure 3:**
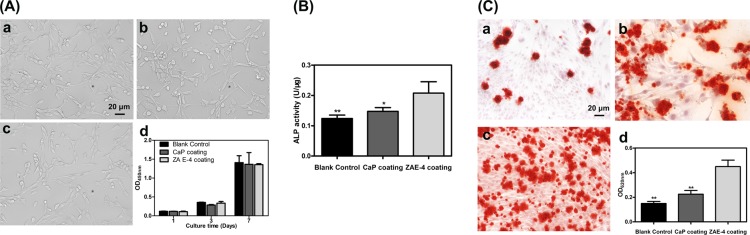
(**A**) The morphologies of MC3T3-E1 cells cultured in different extracts was observed by inverted microscope at day 3, and the images show cells are normal and healthy with typical spindle shape in all group (a) The blank control group; (b) CaP coating; (c) ZA-CaP coating; (d) Cell proliferation assay in different extracts after 1, 3 and 7 days incubation measured by colorimetric CCK-8 assay, and the absorbance of cells in different group is gradually increased. (**B**) ALP activity (U/μg) of MC3T3-E1 cells induced by ZA-CaP bilayer coating Mg-Sr alloy is clearly elevated and significantly higher than other groups at 7 d (p < 0.01). (**C**) The extracellular calcium deposition of MC3T3-E1 cells in ZA-CaP bilayer coating Mg-Sr alloy group is stained intensively and increased visibly (a) The blank control group, (b) CaP coating, (c) ZA-CaP coating, (d) The semiquantitative analysis of calcium nodule). *p < 0.05 and **p < 0.01 compared with ZA-CaP bilayer coating Mg-Sr alloy.

To investigate the effects of Mg and Sr ions, as well as ZA released from our Mg-1.5%Sr alloy on the cyto-compatibility of pre-osteoblasts MC3T3-E1, cells are treated with different Mg-Sr alloy extracts. Figure 3A shows that the morphologies of MC3T3-E1 cells cultured in different extracts are healthy and uniform with typical spindle shape at day 3, which is similar to that of the blank control. CCK-8 assay (Fig. 3A–d), as an indicator of the dehydrogenases in viable cells, shows that the absorbance values of MC3T3-E1 cells treated with Mg-Sr alloys are also relatively similar to the blank control within 7 days, and the difference is not significant (p > 0.05). Additionally, the relative growth rate of different groups increases continuously, therefore, cell numbers are increased with the extension of incubation.

### Intracellular alkaline phosphatase (ALP) activity and extracellular matrix mineralization of pre-osteoblasts

The intracellular ALP activity is recognized as a typical early marker of osteogenic differentiation. We evaluate the ALP activity (U/μg) of MC3T3-E1 cells by standardizing to the contents of intracellular total protein at 7 days after osteogenic induction by Mg-Sr alloys extracts. As shown in Fig. 3B, the ALP activity of MC3T3-E1 cultured in ZA-CaP bilayer coating Mg-Sr alloy extracts reaches the highest value at day 7 (p < 0.01), however, the difference between CaP monolayer coating Mg-Sr alloy and the blank control is not significant (p > 0.05). The order from highest to lowest of the ALP activity is: ZA-CaP bilayer coating Mg-Sr, CaP monolayer coating Mg-Sr, and the blank control.

The bio-mineralization, as a classical late marker of osteogenesis, is evaluated by the deposition of calcium through Alizarin Red S staining at 14 days after osteogenic induction by Mg-Sr alloys extracts. As shown in Fig. 3C, more mineral nodules and intensive staining of the extracellular matrix mineralization is present in ZA-CaP bilayer coating Mg-Sr alloy than others. In the quantitative results (Fig. 3C–d), the relative value of extracellular matrix mineralization induced by CaP monolayer coating and ZA-CaP bilayer coating Mg-Sr alloy enhances by 1.5-times and 3-times relative to the blank control, respectively, which indicated the significant acceleration of bio-mineralization by our surface modified Mg-Sr alloys.

### ZA-CaP bilayer coating Mg-Sr induced apoptosis of pre-osteoclasts

**Figure 4:**
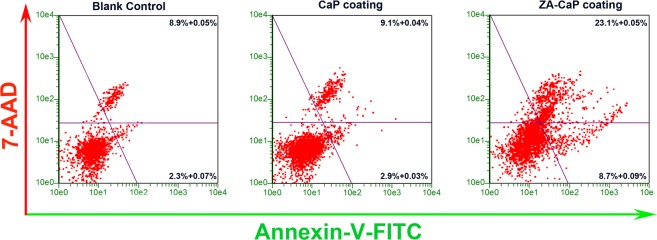
Apoptosis of pre-osteoclasts RAW264.7 after being cultured with different Mg extracts for 24 h. The images show that the apoptosis and necrosis cells of ZA-CaP bilayer coating Mg-Sr alloy reached to 8.7% and 23.1%, respectively, which are significantly higher than other groups (p < 0.01).

The apoptosis of osteoclasts induced by Mg-Sr alloys is quantified by flow cytometry analysis through a fluorescence double-dye. Briefly, Annexin V-FITC binds to the phosphatidylserine exposed on the surface of cells undergoing apoptosis, and 7-AAD labels the late-stage apoptotic and necrotic cells. As shown in Fig. 4, flow cytometry scatter plots suggest that there is no obvious apoptosis (Annexin V-FITC-positive and 7-AAD-negative cells) and necrosis (Annexin V-FITC-positive and 7-AAD-positive cells) in CaP monolayer coating Mg-Sr alloy and blank control, and the percent of healthy cells is greater than 85%. However, ZA-CaP bilayer coating Mg-Sr alloy results in a dramatic increase of the early apoptosis to 8.7%, and the necrosis to 23.1% (p < 0.01). These results confirm that ZA released from Mg-Sr alloy could selectively and strongly induce the apoptosis and necrosis of pre-osteoclasts.

### ZA-CaP bilayer coating Mg-Sr inhibited cytoskeleton organization of pre-osteoclasts

**Figure 5:**
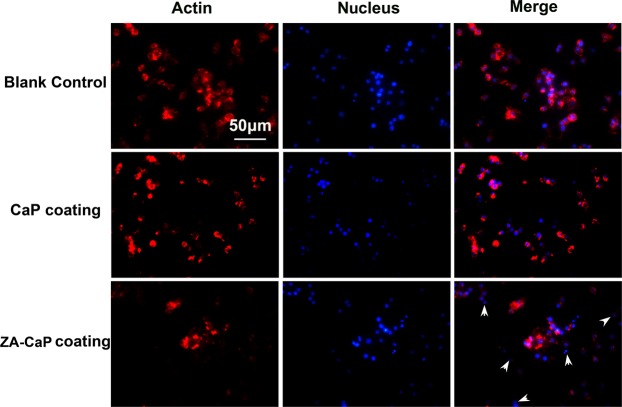
Cytoskeleton organization of pre-osteoclasts RAW264.7 after being cultured with different Mg alloy extracts for 24 h. Cells are stained to visualize F-actins (red) and cell nuclei (blue), and ZA-CaP bilayer coating Mg-Sr alloy gets low densities, and exhibited less spreading morphology, more immature skeleton and weaker focal adhesion (white arrows) in the fluorescence micrographs.

Actin mediates and stabilizes the linkage between the integrin and cytoskeleton, which further control the cell adhesion and cell moility^18^. The cytoskeleton actin-cell nucleus bi-color staining images (Fig. 5) show that worse and thinner organized filamentous actin bundles, and significantly smaller area of individual osteoclasts (white arrows) are observed on ZA-CaP bilayer coating Mg-Sr compared to CaP monolayer coating Mg-Sr and the blank control, which indicates unfavorable adhering and spreading properties of pre-osteoclasts exposed to specified ZA-CaP bilayer coating Mg-Sr alloy.

### ZA-CaP bilayer coating Mg-Sr suppressed osteoclastogenesis

**Figure 6:**
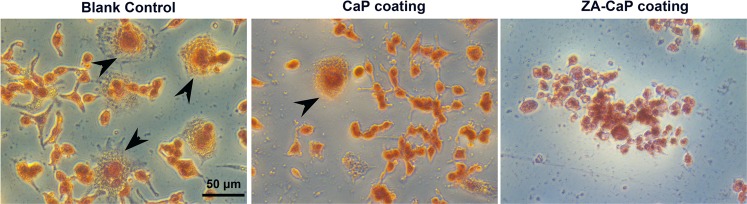
ZA-CaP bilayer coating Mg-Sr alloy inhibits RANKL-induced osteoclast formation. Pre-osteoclasts are treated with different Mg alloy extracts followed by osteoclastogenesis revulsant stimulation for 7 days. Cells are subjected to TRAP staining and the TRAP-positive multinuclear cells (black arrows) are obvious in the blank control and CaP monolayer coating Mg-Sr group.

To investigate the effect of ZA released from Mg-Sr alloy on osteoclastogenesis, pre-osteoclasts are treated with ZA-CaP bilayer coating Mg-Sr during osteoclast formation. Within the osteoclastogenesis a re-organization of the actin cytoskeleton takes place and osteoclast characteristic structures like actin rings are developed. As shown in Fig. 6, there are numerous tartrate-resistant acid phosphatase (TRAP)-positive multinucleated osteoclasts (black arrows) formed in the blank control group. By contrast, the numbers of multinucleated osteoclasts are strikingly decreased in CaP monolayer coating and ZA-CaP bilayer coating Mg-Sr alloy group, indicating the maturation of osteoclasts is significantly suppressed. In particular, the osteoclasts in ZA-CaP bilayer coating group are approximately 90% less than the blank control group. Collectively, these results suggest ZA released from the Mg-Sr alloy plays a dominant role in the inhibiting of osteoclastogenesis.

### ZA-CaP bilayer coating Mg-Sr enhanced ERα activity of pre-osteoclasts

**Figure 7:**
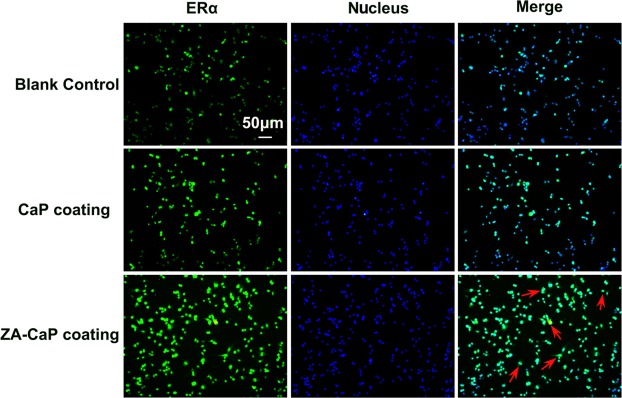
ZA-CaP bilayer coating Mg-Sr alloy treatment triggers the activation of ERα on pre-osteoclasts. RAW264.7 cells are pre-treated with Mg alloy extracts followed by osteoclastogenesis revulsant stimulation for 7 days. Immunofluorescent analyses show that the level of ER alpha in the osteoclast treated with ZA-CaP coating Mg-Sr alloy is highly up-regulated compare to other two conditions.

Previous studies have demonstrated that the imbalance of bone remodeling process induced by excessive bone absorption could be mediated by ERα activity^19^. Therefore, regulating the activity of ERα could be used as a new treatment strategy for periprosthetical osteolysis. To determine whether ERα is involved in down-regulated osteoclastogenesis in the presence of ZA released from Mg alloys, the immunofluorescence staining is conducted, and we found that the expression of fluorescently-labeled ERα on the pre-osteoclasts membrane is significantly enhanced by ZA-CaP bilayer coating Mg-Sr alloy compare to other two conditions (Fig. 7, red arrows), which may prevent continuous osteoclastogenesis process.

### ZA-CaP bilayer coating Mg-Sr down-regulated the expression of osteoclastogenesis-related genes and proteins

**Figure 8:**
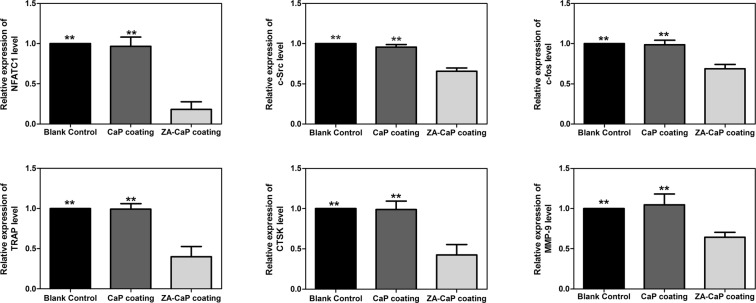
Relative expression of top differentially expressed genes in pre-osteoclasts induced by Mg-Sr alloy extracts containing osteoclastogenesis revulsant for 7 days using qRT-PCR. In each case, data are normalized to the expression level of β-actin. The average mRNA expression levels of ZA-CaP bilayer coating Mg-Sr alloy are markedly down-regulated. **p < 0.01 compared with ZA-CaP bilayer coating Mg-Sr alloy.

**Figure 9:**
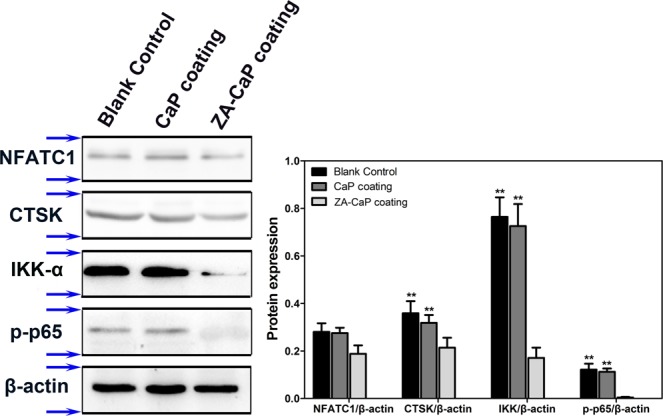
The expression of typical proteins involved in the osteoclastogenesis by RAW264.7 after incubation for 7 days in Mg alloy extracts. Total proteins are extracted from RAW264.7 cells and analyzed by Western blotting. Expression of β-actin is used as an internal control. The semi-quantitative analysis and corresponding histogram indicate that ZA coating Mg-Sr alloy could decrease the NFATC1, CTSK, IKK-α and p-p65 protein expression (p < 0.01). Blots for different proteins were cropped and vertically stacked into one image with white area separated in between different proteins. Blue arrows indicate the horizontal cropping lines. All gels were run under the same experimental conditions. ^##^p < 0.01 compared with CaP coating Mg-Sr alloy. **p < 0.01 compared with blank control.

The differentiation and maturation of osteoclasts is regulated by the expression of osteoclastogenesis-related genes and proteins, such as TRAP, c-Src, Central transcriptional factor in osteoclastogenesis (c-fos), Cathepsin K (CTSK) and Matrix metalloprotease-9 (MMP-9), most of which are targets of Nuclear factor of activated T-cells cytoplasmic 1 (NFATC1). For the purpose of further investigating the molecular mechanisms of Mg-Sr alloy affecting osteoclastogenesis, qRT-PCR is conducted after 7 days culture. Our results show that the mRNA expression level of osteoclastogenesis-specific genes decrease significantly in ZA-CaP bilayer coating Mg-Sr (Fig. 8, p < 0.01), however, the difference between CaP monolayer coating and blank control is not significant (p > 0.05). Similar to the gene expression, ZA-CaP bilayer coating Mg-Sr also inhibits the protein expression of osteoclastogenesis-related NFATC1 and CTSK, as well as IKK-α and phosphorylated p65 of NF-κB signaling pathway (Fig. 9). These results support the idea that ZA-CaP bilayer coating Mg-Sr alloy inhibits osteoclastogenesis and bone resorption through down-regulating the expression of osteoclastogenesis-specific biomarkers and suppressing the activation of NF-κB signaling pathway.

### ZA-CaP bilayer coating Mg-Sr regulated the production of cytokine in pre-osteoblasts and pre-osteoclasts co-culture system

**Figure 10:**
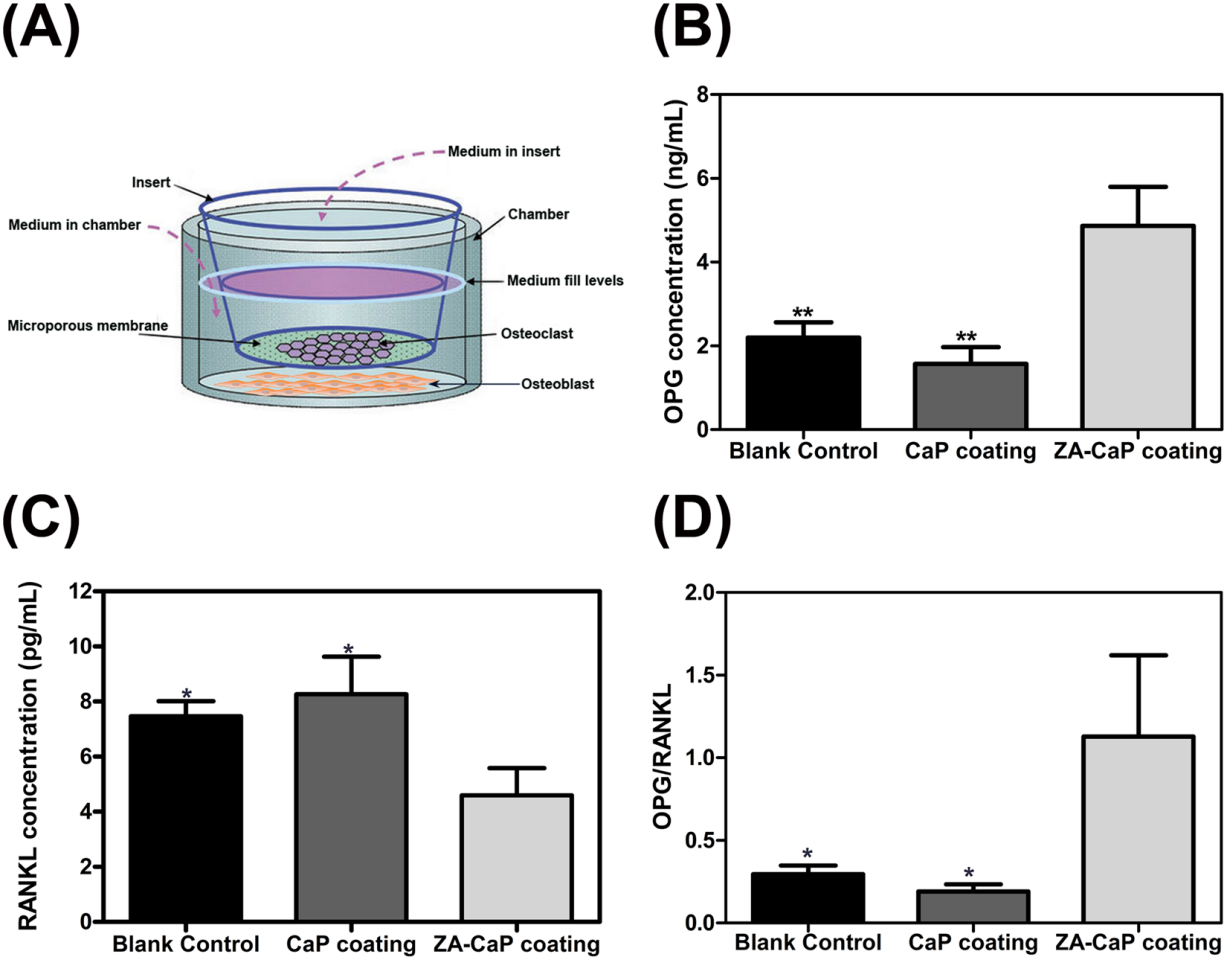
Regulation of OPG and RANKL production in MC3T3-E1 cells by Mg-Sr alloy under the co-culture of osteoblasts with osteoclasts. (**A**) Schematic representation of the experiment. (**B**) The secretion of OPG proteins detected by ELISA. (**C**) The secretion of RANKL proteins detected by ELISA. (**D**) The ratio of OPG/RANKL. **p < 0.01 compared with ZA-CaP bilayer coating Mg-Sr alloy.

Bone tissue comprises the dynamic crosstalk of osteoblasts and osteoclasts for bone formation and resorption^20^, and co-cultures represent a superior model for mimicking natural bone microenvironment^21^. Figure 10 illustrates the effect of Mg alloy on the production of OPG and RANKL signaling proteins known to modulate osteoclast differentiation and activity. Results indicate that ZA-CaP bilayer coating Mg-Sr alloy could regulate the cellular communication between osteoblasts and osteoclasts, and increase the propotion of OPG:RANKL secreted from osteoblasts in transwell co-culture system compared to CaP monolayer coating Mg-Sr and the blank control (Fig. 10B p < 0.05), which is due to the increases in OPG (p < 0.05) and the decreases in RANKL (Fig. 10C, p < 0.01). Because of the relative expression of OPG and RANKL in osteoblasts is a critical transition point for balancing bone mineralization^22^, ZA-CaP bilayer coating Mg-Sr alloy is more than likely balancing osteoblast and osteoclast activities to maintain appropriate bone remodeling.

## Discussion

It has been well established that the reconstruction and remodeling process of bone defects involves close communication between osteoblasts and osteoclasts. These cells work together in a tightly regulated cycle in order to maintain bone tissue structural integrity^23^. However, the long term effectiveness of bone implants is limited by the chronic inflammation induced by the wear-debris, and periprosthetical osteolysis is still the most frequent complication occurring in patients with osteoclastic bone resorption^24,25^.

Mg-based biomaterials for the treatment of bone defects have attracted increasing attentions because of their biodegradability, good biocompatibility and mechanical properties^26^. However, further optimization of their constitution and surface modifications are necessary to make them biologically and physicochemically more suitable for medical applications. Numerous studies and our previous researches have indicated that Mg-Sr alloys exhibited prominent advantages as bone substitutes, such as the satisfied mechanical properties and osteo-promotion performance, as well as the tailored degradation rates by coating modification^27,28^. In addition, ZA pretreatment could enhance the proliferation and osteogenesis without impairment of BMSC immunomodulatory properties *in vitro*^29^. Different graft materials loaded with ZA could be considered as available therapeutic method to improve the bone formation process in the calvarial bone defects *in vivo*^30^. Therefore, in the present study, our objective was to develop and characterize a ZA-CaP bilayer coating Mg-Sr alloy formed by depositing ZA on the alloy surface through the strong ionic interaction between Ca^2+^ and the negative charges^31^, and then evaluate the influence of ZA coating on the osteogenesis, osteoclastogenesis and osteoblast-osteoclast cross talk. For the first time, we proposed that ZA coating Mg-Sr alloy could simultaneously induce osteogenesis and inhibit osteoclastogenesis through the effects of ZA released from Mg-Sr alloy. Although the release profile of ZA only lasts for 4 days, the dose range is appropriate for the positive effect in bone formation. In addition, ZA release could also accommodate the biological process of bone remodeling through regulating the osteo-immunology and dampening the activity of osteoclasts at the early period of implantation, and then reach a persistent equilibrium, thus avoiding osteoclasts reactivation. Meanwhile, we demonstrated that ZA-CaP bilayer coating Mg-Sr alloy inhibited the NF-κB signaling pathway through suppressing the activation of IκB kinase α(IKK-α) and the phosphorylation of p65 subunit of NF-κB in osteoclasts, which was consistent with previous reports^32^.

Effective osseointegration of orthopedic implants requires a sequential process: the recruitment of mesenchymal stem cells or osteoprogenitor cell to the surface of bone implants, the formation of new bone matrix following osteogenic differentiation and mineralization, and bone remodeling^33^. High ALP activity and calcified extracellular matrix are two representative indexes closely related to the osteoblastic phenotype, which pronouncing the maturation of osteoblast^34^. In this context, we demonstrate that ZA-CaP bilayer coating Mg-Sr alloy could significantly promote the differentiation and mineralization of pre-osteoblasts, indicating the acceleration for osseointegration process under the bone microenvironment. Besides, Osteoclast activity is tightly coupled to osteoblast activity under physiological conditions because of the dynamic interactions between osteoblasts and osteoclasts for bone formation and bone resorption^20^, and the property of bone graft substitutes not only depends on the osteoblasts behaviors but also on the osteoclast activities, with their mutual effects, influence the procedure of bone remodeling. The differentiation of the pre-osteoclasts exposed to different materials is supported by the multinuclearity in their morphology and the intracellular TRAP synthesis, as well as the modulation of cytoskeleton organization^35^. We observed that the actin cytoskeleton of pre-osteoclast was irregular and the TRAP activity was significantly down-regulated under the treatment of ZA-CaP bilayer coating Mg-Sr alloy. TRAP-negative was also confirmed by qRT-PCR (Fig. 8), indicating the phenotype of osteoclastogenesis was significantly inhibited by the bilayer coating modified Mg-Sr alloy.

The coupling of osteoblasts and osteoblasts constitutes the complex process of bone reconstruction. Indeed, understanding the role of osteoclasts is no longer limited to osteoclastogenesis, but also having a role on the osteoblasts proliferation, differentiation and maturation^36^. Therefore, the co-culture systems with bio-fabricated constructs were utilized to mimic natural tissues in their full complexity in our research (Fig. 10A), and the ratio of OPG/RANKL, as a critical factor determining bone rebuilding/bone resorption (i.e., ratio >1 pro-osteogenesis and ratio <1 pro-osteoclastogenesis), was detected. OPG is secreted by osteoblasts and blocks the binding of RANKL to RANK through acting as a competitive inhibitor of RANK receptor, thereby attenuating excessive RANKL signaling^37^. As shown in Fig. 10D, the ratio of OPG/RANKL was more than 1 in our co-culture system, indicating the acceleration of bone healing.

The processes of bone remodeling are regulated by estrogen, OPG/RANKL/RANK and other related kinase signal transduction pathways^38^. ERα is highly expressed throughout the osteoblast developmental sequence and plays a positive role at both proliferation and differentiation stages during bone formation, and blocking the expression of ERα by treatment with antisense oligonucleotides or siRNA leads to decreased bone nodule formation^39^. Our immunofluorescence results showed that ZA-CaP bilayer coating Mg-Sr alloy treatment could significantly increase the expression of ERα on osteoclasts through the fluorescence intensity enhanced. OPG/RANKL/RANK signaling pathway modulates osteoclast survival through the binding of ligands (RANKL) to receptors (RANK) on the surface of osteoclast precursors, and then activates the transcriptional factors (c-fos, NFATC1 and c-Src) and osteoclastogenic genes (CTSK and MMP-9) responsible for the survival and differentiation of osteoclasts^10,40^. NFATC1 regulates the expression of numerous osteoclasogenic genes, and it has been reported that embryonic stem cells with NFATC1-genetic deficiency cannot differentiate into mature osteoclasts^41^. c-Src influences bone absorption through mediating the adhesion and migration of osteoclasts^42^. CTSK and MMP-9 involve in the degradation of bone matrix and then induce the activation of bone-resorptive^43^. Our qRT-PCR and Western blotting results showed that ZA-CaP bilayer coating Mg-Sr alloy suppressed osteoclastogenesis by down-regulating the expression of these important osteoclast-specific factors.

We check the protein expression level of NF-kB signaling pathway because that it has been established that the transcriptional activity of NF-κB signaling pathway mediates RANKL-associated osteoclastogenesis. The binding of RANKL to RANK induces a series of cascade reactions of NF-κB, including the activation of IKK through the complex formation and the phosphorylation of inhibitory protein IκB, which trigger the accumulation and translocation of NF-κB subunit p65/RelA into the cell nucleus. Then, the osteoclastogenesis-related target genes, such as NFATC1, are activated^44^. Our results showed that ZA-CaP bilayer coating Mg-Sr alloy could dramatically suppress the activation of NF-κB signaling pathway by the inactivation of IKK and tyrosine phosphorylation of p65/NF-κB. These findings provided support for the idea that NF-κB signaling pathway was essential for the therapy mediated by our surface modified Mg-Sr alloys.

Further, it is important to highlight that our *in vitro* study is only the first characterization of this Mg-Sr system for the delivery of ZA. Subsequent *in vivo* studies and final clinical assay should be conduct to evaluate the effectiveness of this system that acts as bone grafting for *in situ* administration and controlled release of loaded ZA, the conservation of implants bioactivity, the ZA distribution zone, and the occurrence of possible undesirable effects. Finally, clinical assays should evaluate the performance of this system if possible.

## Conclusions

**Figure 11:**
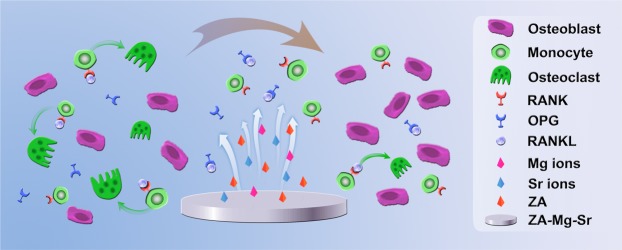
Schematic illustration of ZA-CaP bilayer coating Mg-Sr alloy regulating the cross-talk of osteoblast-osteoclast in the microenvironment of bone remodelling through OPG/RANKL/RANK signalling pathway. ZA and ions released from the Mg-Sr alloy could promote the secretion of OPG from osteoblasts, which competitively interrupt the RANK-RANKL system. Therefore, our surface modified Mg-Sr alloy could inhibit the osteoclasts differentiation from monocytes and osteoclast maturation, as well as be beneficial to osteogenesis.

A novel bilayer coating (ZA-CaP) has been fabricated on Mg-Sr alloy by the marked affinity between zoledronic acid and Ca^2+^ by chemical deposited CaP layer coating. We have demonstrated the ZA-CaP bilayer Mg-Sr alloy for the bone grafting application. It’s beneficial for this substitute without any signs of cytotoxicity and harmful effects on osteoblast proliferation, however inducing excellent osteogenic effects. We also indicate that ZA-CaP bilayer coating Mg-Sr alloy could synergistically induce apoptosis and inhibit osteoclast differentiation of pre-osteoclasts through the enhancement of ERα activity and the blocking of NF-κB signaling pathway. Moreover, ZA-CaP bilayer coating Mg-Sr alloy could regulate the cross-talk of osteoblast-osteoclast under cell co-culture system through OPG/RANKL/RANK pathway as shown in Fig. 11. Taken together, our results provide evidence that ZA-CaP bilayer coating Mg-Sr alloy may represent a new local adjuvant therapy for bone defect repair in future.

## Methods

### Materials preparation and characterizations

In this work the Mg-1.5wt.% Sr alloys were used as substrates to prepare ZA-loaded CaP coating^45^. For the preparation of CaP coating, samples were immersed in 0.1 M KF for 24 h, and subsequently soaked in the mixed solution of 60 g/L NaNO_3_, 15 g/L Ca(H_2_PO_4_)_2_·H_2_O and 20 mL/L H_2_O_2_ for 24 h. Afterwards, ZA loaded Mg-Sr alloys were produced by soaking the implants in 5 mL of aqueous ZA solution (10^−4^ mol/L) at 37 °C for 24 h through the chemical association of ZA with CaP coating. Samples were then cleaned with deionized water and dried with a gentle stream of dry air (termed ZA-CaP bilayer coating Mg-Sr alloy).

The morphologies of the surface-modified Mg-Sr alloys were observed under a scanning electron microscope (SEM, S-3400N, Hitachi). The crystalline phases were identified by an XRD (Rigaku DMAX2500) using CuKα radiation at a scan speed of 0.05 °/min in 2θ with 1 ° as incident beam angle.

The release of ZA from surface modified Mg-Sr alloy was measured by High-Performance Liquid Chromatography (HPLC, e2695, Waters)^46^. Primary stock standard solutions of ZA (500 μg/mL) was prepared and then diluted with deionized water to obtain a series of working solutions (0.2, 0.5, 2, 5, 20, 50, 200 and 500 μg/mL). The concentration of aqueous ZA solutions was determined by directly adding the samples into the HPLC system with the volume of 50 μL. Chromatographic separation was achieved with an Agilent XDB-C18 column (150 mm × 4.6 mm). The fluid phase comprised acetonitrile and phosphate buffer (2:8, v/v), and the monitored wavelength was 220 nm.

### Cell preparation and culture

The murine calvarial pre-osteoblast MC3T3-E1 (ATCC, CRL-12424) and murine pre-osteoclast RAW264.7 (ATCC, SC-6005) were incubated in α-MEM (Gibco) and high glucose DMEM (Gibco) containing 10% FBS (Gibco) at 37 °C in a humidifed atmosphere of 5% CO_2_ routinely, respectively. To evaluate interaction between Mg alloys and cells, Mg-Sr alloy extracts were prepared using cell culture medium (α-MEM or DMEM) containing 10% FBS with an extraction ratio of 1.25 cm^2^/mL at 37 °C for 24 h. Osteogenesis of pre-osteoblasts was induced by the Mg-Sr alloys extracts supplemented with 0.01 μM dexamethasone, 10 mM Na-β-glycerophosphate and 50 ug/mL ascorbic acid (Sigma). Osteoclastogenesis of pre-osteoclasts was induced by the Mg-Sr alloys extracts supplemented with 50 ng/mL RANKL (R&D system).

### Osteoblasts proliferation assay

Pre-osteoblasts MC3T3-E1 cells were seeded into 96-well plates with a density of 1 × 10^4^ cells per well and incubated for 24 h to allow attachment. Then the Mg-Sr alloy extracts were used to replace cell culture medium. Osteoblasts cultured with α-MEM containing 10% FBS was set as the blank control. Cell proliferation was determined with CCK-8 assays (DOJINDO) after incubation for 1, 3, and 7 days as we described previously^6^.

### Intracellular ALP assay

MC3T3-E1 cells with a density of 1 × 10^4^ cells per well were treated with Mg-Sr alloy extracts supplemented with osteogenesis revulsant. Cells cultured with normal medium supplemented with osteogenesis revulsant were set as the blank control. After 7 days of culture, cells were lysed and the intracellular ALP activities were measured by catalysing the p-nitrophenyl phosphate (p-NPP, Sigma) substrate to produce a chromogenic reaction. The activities were ultimately normalized to the intracellular protein.

### Extracellular matrix mineralization

MC3T3-E1 cells with a density of 5 × 10^4^ cells per well were treated with Mg-Sr alloy extracts supplemented with osteogenesis revulsant for 14 days and Cells cultured with normal medium supplemented with osteogenesis revulsant were set as the blank control. Then the mineralization of extracellular matrix was stained with Alizarin Red solution (40 mM, pH 8.3, Sigma). Afterwards, cells were washed until the absence of color, and the mineralization staining were observed by microscope (Leica). For quantitative analysis, the staining nodules were dissolved in sodium phosphate (10 mM, sigma) and measured at 620 nm.

### Osteoclasts apoptosis assay

The apoptotic effect of ZA-CaP bilayer coating Mg-Sr alloy on pre-osteoclasts RAW264.7 was evaluated by using Guava Nexin Reagent (Millipore). Briefly, 1 × 10^5^ cells were treated with Mg-Sr alloy extracts for 24 h and re-suspended in 100 μL DMEM containing 1% FBS. Cells treated with DMEM containing 10% FBS was set as the blank control. Early apoptotic/necrotic cells were stained by 100 μL Annexin V-FITC/7-AAD labelling solution for 20 min at 37 °C. The fluorescence-activated cells were then evaluated by flow cytometer (Millipore).

### Cytoskeleton organization observation

Cytoskeletal changes of pre-osteoclasts were performed by using a double-dye staining (Millipore) of the actin and cell nucleus. Untreated cells adhered on 24-well plates were set as the blank control, after co-culture for 24 h, RAW264.7 cells adhered on the Mg-Sr alloy samples were fixed by 4% paraformaldehyde (PFA) for 10 min, followed by permeabilization using 0.1% Triton X-100. Cell cytoskeletal actin and nucleus was stained by Tetramethylrhodamine isothiocyanate (TRITC)-conjugated phalloidin (37.5 ng/mL, ThermoFisher scientific) for 1 h and 4′,6-diamidino-2-phenylindole (DAPI, 10 μg/mL, Sigma) for 10 min, respectively. Finally, fluorescence staining images were obtained by the laser scanning confocal microscopy (LSM 700, ZEISS).

### TRAP activity

RAW264.7 cells seeded in 96-well plates at 4 × 10^3^ cells per well were treated with Mg-Sr alloy extracts supplemented with osteoclastogenesis revulsant for 7 days, and cells cultured with normal medium supplemented with osteoclastogenesis revulsant were set as the blank control. Cells were then fixed by 4% PFA at room temperature for 10 min. The TRAP activity of osteoclasts was assessed by staining with an acid phosphatase kit (Sigma). The staining results were evaluated microscopically (Leica) and TRAP-positive multinucleated cells were indicated.

### Immunofluorescence staining

The expression of estrogen receptor α (ERα) in pre-osteoclasts RAW264.7 was detected by immunofluorescence labelled. Briefly, RAW264.7 cells were fixed with 4% PFA after exposing to Mg-Sr alloys extracts for 24 h and washed three times with PBS. Permeabilized treatment is necessary to osteoclasts by using 0.1% Triton X-100 solution at room temperature for 10 minutes. To block the nonspecific-binding, cells were incubated with blocking buffer (1% BSA) for 1 h. Primary antibody against ERα (1:1000, CST) was added and reacted with cells at 4 °C overnight, followed by incubating with goat anti-Rabbit secondary antibody labelled with Alexa Fluor 488 (1:1000, Thermofisher scientific) for 1 h and DAPI (10 μg/mL, sigma) at room temperature for 5 min. The immunofluorescence staining of ERα protein (green) and cell nucleus (blue) could be simultaneously viewed by a laser confocal microscope (LSM 700, ZEISS) at an excitation wavelength of 405 nm and 488 nm, respectively. Next, the green and blue images were overlaid and the co-localization area was produced in cyan fluorescence.

### qRT-PCR

RAW264.7 cells were seeded at 1 × 10^5^ cells/well into 24-well plates and treated with Mg-Sr alloys extracts supplemented with osteoclastogenesis revulsant for 7 days. TRIzol reagent (Thermo Fisher Scientific) was used to extract the intracellular total RNA and cDNA was immediately synthesized by using reverse transcriptase (TaKaRa). qRT-PCR was performed using SYBR qPCR Mastermix (Bestar) and Rotor gene Q Sequencing Detection System (Qiagen). The PCR amplification was performed with the following program: 95 °C for 15 min, followed by 40 cycles including 95 °C for 10 s and 60 °C for 30 s. All results were calculated using 2^−ΔΔct^ method as previous described^47^, and we set the β-actin as the reference gene to standardize the calculation. The mouse primer sequences were present as below: NFATC1 (5′-AGCAGAGCACGGACAGCTATC-3′ and 5′-AGGTCCCGGTCAGTT TTCG-3′); TRAP (5′-TACCCCGTGTGGTCCATAGC-3′ and 5′-GTAGCCCACGC CATTCTCAT-3′); CTSK (5′-CCATATGTGGGACAGGAAGAGAGT-3′ and 5′-TG CATCAATGGCCACAGAGA-3′); c-Src (5′-CCAGGCTGAGGAGTGGTACT-3′ and 5′-CAGCTTGCGGATCTTGTAGT-3′); c-fos (5′-TCCGAAGGGAAAGGAAAAGA TG-3′ and 5′-TTTCCTTCTCCTTCAGCAGGTT-3′); MMP-9 (5′-CCTGGAGACCT GAGAACCAATC-3′ and 5′-GTCTCGGGCAGGGACAGTT-3′) and β-actin (5′-GG GAAATCGTGCGTGACATT-3′ and 5′-GGAACCGCTCATTGCCAAT-3′).

### Western blot analysis

RAW264.7 cells were seeded in 24-well plates with a density of 1 × 10^5^ cells/well and treated with different Mg alloy extracts supplemented with osteoclastogenesis revulsant for 7 days. Cells cultured with DMEM containing 10% FBS was set as the blank control. The intracellular protein content was detected by using BCA protein assay kit (Pierce) after lysing with Radio-Immunoprecipitation lysis buffer (Beyotime) according to the manufacture’s protocol. Electrophoretic analysis of protein lysate (40 μg) were carried out on 10% sodium dodecyl sulphate-polyacrylamide gel electrophoresis (SDS-PAGE), and immunoblot analysis were performed on polyvinylidene difluoride (PVDF) membranes (Millipore). Non-specific binding were removed by blocking buffer (1% BSA) for 1 h. The corresponding bands positively reacted with specific anti-NFATC1 (1:1000, CST), -CTSK (1:500, abcam), -IKK-α(1:10000, abcam), -phospho-NF-κB p65 (1:1000, CST) and -β-actin (1:2000, CST) at 4 °C overnight, and then with secondary antibodies (1:10000, ThermoFisher Scientific) labelled with horseradish peroxidase (HRP) at 37 °C for 1 h. The chemiluminescence detection method (Millipore) and ChemiDoc MP imaging system (Bio-Rad Laboratories) were then used to visualize and scan the signals of immunoblots, respectively.

### Co-culture of pre-osteoblasts with pre-osteoclasts and cytokine production analysis

Transwell chamber culture system (Corning life sciences) was used to evaluate the intercellular contact of pre-osteoblasts with pre-osteoclasts. 5 × 10^4^ RAW264.7 cells were seeded into the upper chambers, which had a layer of polycarbonate membrane with 0.4 μm pores on the bottom, and 5 × 10^4^ MC3T3-E1 cells were cultured in the lower chambers of the transwell systems. After 24 h of attachment, MC3T3-E1 cells in the lower chambers were cultured with Mg alloys extracts supplemented with osteogenesis revulsant and RAW264.7 cells in the upper chambers were incubated with Mg alloys extracts supplemented with osteoclastogenesis revulsant. Following 7 days of co-culture, the culture supernatant of osteoblasts was collected and screened for osteoblasts-derived cytokines using OPG and RANKL enzyme-linked immunosorbent assay (ELISA) kit (R&D system) according to the manufacturer’s instructions.

### Statistics

SPSS 13.0 was used to test the significant difference between different groups using one-way analysis of variance (ANOVA). Statistically, the difference was termed significant and highly significant when the p-value was less than 0.05 and 0.01, respectively.

## Supplementary information

Supporting Information
