# Supplementary material for: Regulation of osteogenesis and osteoclastogenesis by zoledronic acid loaded on biodegradable magnesium-strontium alloy
Source: Sci Rep. 2019 Jan 30;9:933. doi: 10.1038/s41598-018-37091-8 (PMC6353919; doi:10.1038/s41598-018-37091-8)
Supplement: Supplementary file 1 — Supporting Information [file 41598_2018_37091_MOESM1_ESM.docx]

Regulation of osteogenesis and osteoclastogenesis by zoledronic acid loaded on biodegradable magnesium-strontium alloy

Mei Li^1,2^, Weidan Wang^3,4^, Peng Wan^5,^*, Ke Yang^3^, Yu Zhang^2,^*, Yong Han^1,^*

1. State Key Laboratory for Mechanical Behavior of Materials, Xi’an Jiaotong University, Xi’an 710049, China

2. Department of Orthopedics, Guangdong General Hospital, Guangdong Academy of Medical Sciences, Guangzhou 510080, China

3. Institute of Metal Research, Chinese Academy of Sciences, Shenyang 110016, China

4. University of Chinese Academy of Sciences, Beijing 100049, China

5. School of Mechanical Engineering, Dongguan University of Technology, Dongguan 523808, China

*Correspondence author: Yong Han (yonghan@mail.xjtu.edu.cn); Yu Zhang (luck_2001@126.com); Peng Wan ([pwan@imr.ac.cn](mailto:pwan@imr.ac.cn))

**Ions release**

The CaP coating magnesium-strontium alloys were immersed in the cell culture medium containing 10% FBS at 37°C in a humidified atmosphere of 5% CO_2_ for 5 days with an immersion ratio of 1.25 cm^2^/mL (according to ISO10993) to investigate the in vitro degradation performance. During the immersion tests, the concentrations of Mg and Sr ions were measured by inductively coupled plasma atomic emission spectrometry (ICP-AES) (Z-2000, Hitachi, Japan) after immersion for 1, 3 and 5 days. The extracts after immersion were filtered to remove the precipitations and centrifuged to obtain the supernatants for measurements. Fig.S1 showed the change in concentration of Mg and Sr ions releasing after immersion for 1, 3 and 5 days. It can be seen that the concentration of Mg and Sr ions increased with the extended immersion time.


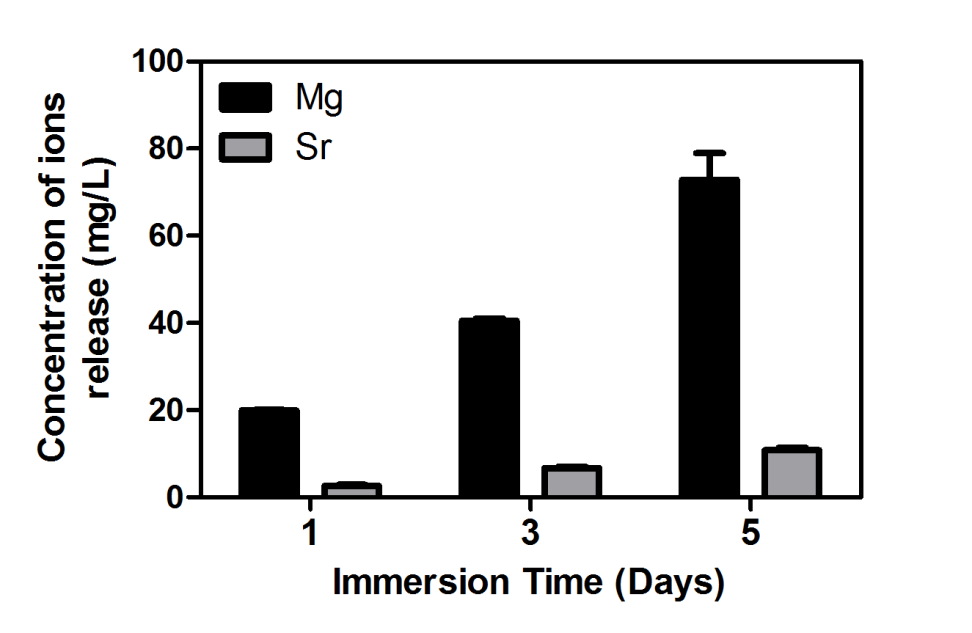


Fig.S1 Mg and Sr ions releasing after immersion of 1, 3, 5 days in the cell culture medium
